# Genome characteristics of *Kordia antarctica* IMCC3317^T^ and comparative genome analysis of the genus *Kordia*

**DOI:** 10.1038/s41598-020-71328-9

**Published:** 2020-09-07

**Authors:** Yeonjung Lim, Ilnam Kang, Jang-Cheon Cho

**Affiliations:** 1 Department of Biological Sciences, Inha University, Incheon, 22212 Republic of Korea; 2 Center for Molecular and Cell Biology, Inha University, Incheon, 22212 Republic of Korea

**Keywords:** Microbial communities, Genome, Microbiology, Bacterial genes

## Abstract

The genus *Kordia* is one of many genera affiliated with the family *Flavobacteriaceae* of the phylum *Bacteroidetes*, well known for its degradation of high molecular weight organic matters. The genus *Kordia* currently comprises eight species, type strains of which have been isolated from a diverse range of marine environments. As of this report, four genome sequences have been submitted for cultured strains of *Kordia*, but none are complete nor have they been analyzed comprehensively. In this study, we report the complete genome of *Kordia antarctica* IMCC3317^T^, isolated from coastal seawater off the Antarctic Peninsula. The complete genome of IMCC3317^T^ consists of a single circular chromosome with 5.5 Mbp and a 33.2 mol% of G+C DNA content. The IMCC3317^T^ genome showed features typical of chemoheterotrophic marine bacteria and similar to other *Kordia* genomes, such as complete gene sets for the Embden–Meyerhof–Parnas glycolysis pathway, tricarboxylic acid cycle and oxidative phosphorylation. The genome also encoded many carbohydrate-active enzymes, some of which were clustered into approximately seven polysaccharide utilization loci, thereby demonstrating the potential for polysaccharide utilization. Finally, a *nosZ* gene encoding nitrous oxide reductase, an enzyme that catalyzes the reduction of N_2_O to N_2_ gas, was also unique to the IMCC3317^T^ genome.

## Introduction

The phylum *Bacteroidetes* is among the most abundant phyla in the ocean, accounting for an average of approximately 10% of marine surface bacterioplankton cells^1,2^. Marine *Bacteroidetes* specializes in the utilization of particulate and high molecular weight organic matter predominately from micro- or macroalgae^3^, and many studies have shown that *Bacteroidetes* increases in abundance during phytoplankton blooms in coastal waters, occupying more than 50% of the bacterial community at certain times^4,5^. Of the many groups within the phylum *Bacteroidetes*, the family *Flavobacteriaceae* is the most abundant in marine environments^4^, and has been subjected to several cultivation and genome-based studies^6–9^. Currently, more than 100 genera with validly published names have been described within the family *Flavobacteriaceae*.

The genus *Kordia* belonging to the family *Flavobacteriaceae* was proposed by Sohn et al.^10^ and currently comprises eight species: *K. ulvae*^11^, *K. algicida*^10^, *K. aquimaris*^12^, *K. zhangzhouensis*^13^, *K. jejudonensis*^14^, *K. periserrulae*^15^, *K. zosterae*^16^, and *K. antarctica*^17^. The type strains of these *Kordia* species have been isolated from a range of marine habitats, including surface seawater, a connection between the ocean and a freshwater spring, seaweed surfaces^16^, and the digestive tract of a marine polychaete. Despite enriched taxonomic diversity and wide distribution, physiological, ecological, and genomic studies on the genus *Kordia* have yet to be performed in-depth beyond those on the algicidal activity of *K*. *algicida* strains^10,18–20^ and a recent report detailing microdiversity among uncultured *Kordia* species identified by single-amplified genomes from a seawater sample of the Indian Ocean^21^.

Currently, whole-genome sequences of four strains of *Kordia* species are publicly available: *K. algicida*, *K. zhangzhouensis*, *K. jejudonensis*, and *K. periserrulae*. However, all of these genome sequences remain in the draft stage, and the only published genome sequence is that of *K. algicida* via a brief single-page genome announcement^22^. Recently, a high-quality, metagenome-assembled genome belonging to the genus *Kordia* was obtained by sequencing a non-axenic culture of the marine diatom *Skeletonema marinoi*^23^. This genome sequence has been described as being acquired from the *Kordia* sp. strain SMS9, but according to reports, attempts to obtain a pure culture of strain SMS9 have been unsuccessful.

This report provides the first complete genome sequence of the genus *Kordia* obtained from *K. antarctica* strain IMCC3317^T^, as well as comparative genome analyses with the four existing genomes of other *Kordia* species. Strain IMCC3317^T^ is a Gram-negative, chemoheterotrophic, yellow-pigmented, non-motile, flexirubin-negative, facultative anaerobic bacterium that was isolated from a coastal seawater sample at the Antarctic Peninsula^17^. To our knowledge, strain IMCC3317^T^ is the only *Kordia* strain isolated from the polar environment thus far. The complete genome sequence of strain IMCC3317^T^ confirmed a chemoheterotrophic lifestyle and showed the potential to utilize polysaccharides and synthesize secondary metabolites. Comparative analyses also indicated that the IMCC3317^T^ genome contains a *nosZ* gene encoding nitrous oxide (N_2_O), unique to this strain among the *Kordia* species.

## Results and discussion

### General genome features

Strain IMCC3317^T^ was isolated from a coastal seawater sample at King George Island in western Antarctica (62°14′ S 58°47′ W) using a standard dilution-plating method and was established as *K*. *antarctica* based on phylogenetic, biochemical, and physiological characterization^17^. General features of strain IMCC3317^T^ are summarized in Baek et al.^17^ and are also presented in Table 1. Comparison of 16S rRNA gene sequences with other type strains of the genus *Kordia* showed that strain IMCC3317^T^ is most closely related to *K. zosterae* ZO2-23^T^ (97.7%, sequence similarity), followed by *K. ulvae* SC2^T^ (97.0%), *K. jejudonensis* SSK3-3^T^ (96.5%), *K. algicida* OT-1^T^ (96.6%), and *K. periserrulae* IMCC1412^T^ (96.1%). Since a complete genome of an axenic strain has yet to be reported for the genus *Kordia*, we derived the complete genome of IMCC3317^T^ using PacBio sequencing.

**Table 1:** General features and genome sequencing information of *Kordia antarctica* strain IMCC3317^T^ according to the MIGS recommendations.

Property	Term
**General features**	
Classification	Domain *Bacteria*
	Phylum *Bacteroidetes*
	Class *Flavobacteriia*
	Order *Flavobacteriales*
	Family *Flavobacteriaceae*
	Genus *Kordia*
	Species *Kordia antarctica*
	Strain: IMCC3317^T^ (= KCTC 32292^T^ = NBRC 109401^T^)
Gram stain	Negative
Cell shape	Rod
Motility	Non-motile
Temperature range; optimum	4–42 °C; 20 °C
pH range; optimum	6.0–9.0; 8.0
Salinity	0.5–5.0% NaCl (w/v)
Oxygen requirement	Facultatively anaerobic
**Investigation**	
Submitted to INSDC	CP019288 (GenBank)
Investigation type	Bacteria_Archaea
Project name	Genome sequencing of *Kordia antarctica* IMCC3317^T^
**Environment**	
Geographic location	King George Island, West Antarctica
Sample collection	2005–2012
Latitude and longitude	62°14′ S, 58°47′ W
Depth	1 m
Environment (biome)	Marine biome (ENVO: 00000447)
Environment (feature)	Coastal water body (ENVO: 02000049)
Environment (material)	Coastal sea water (ENVO: 00002150)
Isolation and growth conditions	PMID: 23606478
**Sequencing**	
Sequencing platform	PacBio RS II (P6-C4 chemistry)
Fold coverage	93.32 ×
Assembly method	SMRT Analysis v.2.3.0
Annotation source	IMG/ER
**Genome features**	
Size	5,500,985
DNA G+C content (%)	33.23
CDS	4,697
rRNA genes	9
tRNA genes	49
CDS assigned to COG	2,057
CDS assigned to Pfam	3,097
CDS assigned to KO	1,408

**Figure 1:**
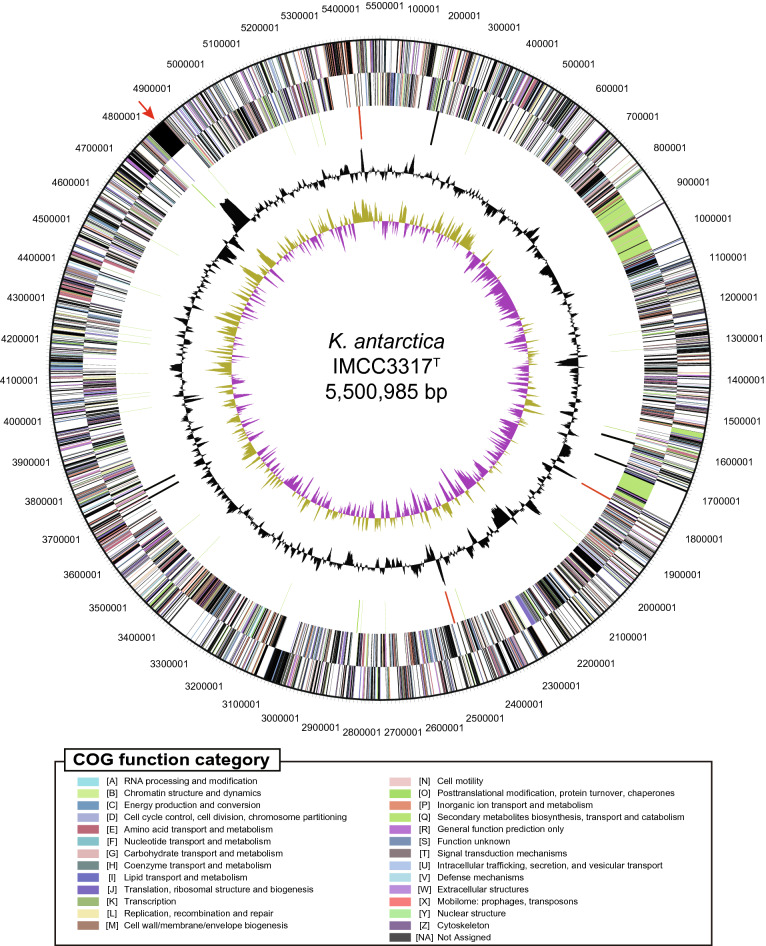
Circular map of the *Kordia antarctica* IMCC3317^T^ genome. From outside to center: Genes on forward strand (colored by COG categories), genes on reverse strand (colored by COG categories). RNA genes: tRNAs, green; rRNAs, red; other RNAs, black. GC content, black; GC skew, purple/olive. Color codes for COG function categories shown below genome map. Position of the ‘giant gene’ indicated by red arrow.

The complete genome of strain IMCC3317^T^ was obtained by de novo assembly followed by five rounds of polishing and comprised a single circular contig. The total length of the genome was 5,500,985 bp and the DNA G+C content was 33.23 mol% (Fig. 1). A total of 4,761 genes were predicted for the genome via the IMG-ER pipeline, including 4,697 protein-coding genes, nine rRNA genes, and 49 tRNA genes. Among the 4,697 protein-coding genes, 62.7% (2,944) could be assigned a putative function. The major COG categories were translation, ribosomal structure and biogenesis (7.74%), coenzyme transport and metabolism (5.97%), and inorganic ion transport and metabolism (5.92%). Overall information on the sequencing and annotation of the IMCC3317^T^ genome is presented in Table 1.

**Figure 2:**
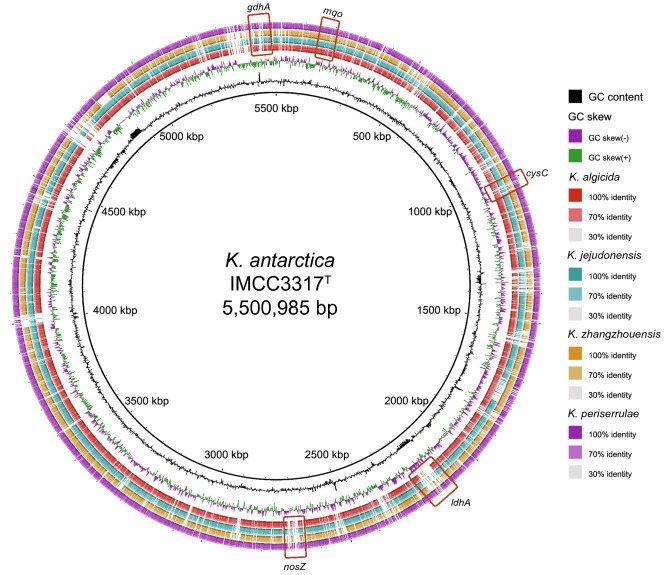
Genomic comparisons among *K. antarctica* IMCC3317^T^ and other *Kordia* strains *K. algicida*, *K. jejudonensis*, *K. zhangzhounensis* and *K. periserrulae*. Innermost rings show G+C content (black) and GC skew (purple/green) of IMCC3317^T^ genome. Other rings show BLASTn-based similarities of IMCC3317^T^ genome to other *Kordia* genomes. Relative shading density within each circle represents level of nucleotide identity. Red boxes indicate the approximate locations of genes unique to the IMCC3317^T^ genome. Abbreviations: *gdhA*, glutamate dehydrogenase (NAD(P)); *mqo*, malate dehydrogenase (quinone); *cysC*, adenylylsulfate kinase; *ldhA*, d-lactate dehydrogenase; *nosZ*, nitrous oxide reductase.

**Figure 3:**
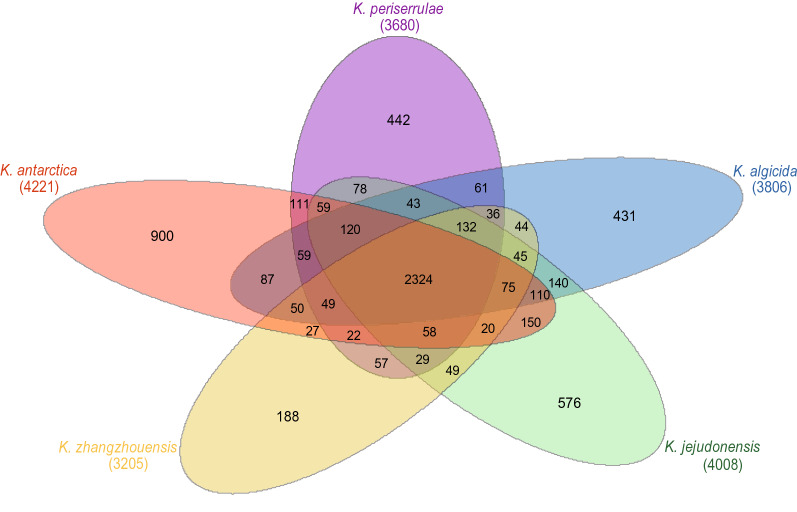
Venn diagram showing number of shared and unique orthologous protein clusters among five *Kordia* strains: *K. antarctica*, *K. periserrulae*, *K. algicida*, *K. jejudonensis*, and *K. zhangzhounensis*. Total number of protein clusters of each genome indicated in parentheses below strain names.

ANI values among the five *Kordia* genomes (including the IMCC3317^T^ genome) ranged from 76 to 80%. These ANI values are lower than 95–96%, a widely accepted threshold for prokaryotic species demarcation^24,25^, and therefore are consistent with the fact that these five genomes are from five different species of the genus *Kordia*. When compared using BLASTn, the IMCC3317^T^ genome showed similarities to the other four *Kordia* genomes over the entire length of the genome (Fig. 2). However, many genomic regions not similar to the four other *Kordia* genomes were also found for IMCC3317^T^. Metabolic genes that were predicted to be unique to the IMCC3317^T^ genome, such as *cysC* and *nosZ* (see below), were found within these regions (Fig. 2). Comparison with other *Kordia* genomes based on the inference of orthologous protein clusters showed that the IMCC3317^T^ genome shared around 55% of its protein clusters (2,324 among 4,221) with all other four *Kordia* genomes, while 900 protein clusters were not found in any other *Kordia* genome (Fig. 3).

A long, 50,835 bp gene was predicted in the IMCC3317^T^ genome. This ‘giant gene’ (IMG gene ID: 2713618241; hereafter, ten-digit numbers in the parentheses following gene or protein names are their IMG gene IDs) had a G+C content of ~ 42 mol% that deviated considerably from the average^26^ (Fig. 1). This gene encoded a protein of 16,944 amino acids in length with more than 100 tandemly-repeated, single Pfam domains (PF03160; Calx-beta). Though a specific function could not be assigned to this protein, the prediction of a signal peptide and a TIGR04131 (gliding motility-associated C-terminal) motif indicated the protein might be involved in cell surface function. BLASTp analysis against the NCBI nr database (accessed at Jul, 2020) showed that many similar proteins are found mostly (but not exclusively) in the family *Flavobacteriaceae*, including other *Kordia* species. The top 10 best hit proteins were also composed of a very large number of amino acids (3,864–12,288), suggesting a wide distribution of long-length proteins similar to the one predicted in the IMCC3317^T^ genome.

### Carbon metabolism

The metabolic capacity of strain IMCC3317^T^ was analyzed using KEGG pathway maps obtained from BlastKOALA and the IMG-ER annotation. The other four *Kordia* genomes available in IMG were also analyzed by BlastKOALA and used for comparative analyses. Since the completeness of other *Kordia* genomes (99.48–99.59%; calculated by CheckM) was only slightly lower than that of the IMCC3317^T^ genome (99.92%), these genome sequences were confident to be used for comparative genome analyses.

Strain IMCC3317^T^ and other four *Kordia* type strains were all predicted to possess complete pathways for central carbon metabolism, such as the Embden–Meyerhof–Parnas (EMP) glycolysis pathway, the tricarboxylic acid (TCA) cycle, and the non-oxidative branch of the pentose phosphate pathway (Table 2). However, strain IMCC3317^T^ was distinct from other strains in the number of enzymes it possessed for several steps in these pathways. While the other four strains encoded but a single copy of 6-phosphofructokinase, one of the most important regulatory enzymes of the EMP pathway, IMCC3317^T^ encoded two copies thereof, with one copy (2713615308) showing more than 90% similarity to homologs from other strains and the other (2713616029) having with less than 50% similarity to these homologs. In addition, while the other four strains each had one class II fructose-bisphosphate aldolase, IMCC3317^T^ encoded the class I fructose-bisphosphate aldolase (2713616027) as well as the class II enzyme. All five strains contained NAD-dependent malate dehydrogenase (EC 1.1.1.37), an enzyme that converts malate to oxaloacetate in the TCA cycle, but strain IMCC3317^T^ additionally harbored a malate dehydrogenase (quinone) (EC 1.1.5.4; 2713614246), a membrane-associated enzyme that can donate electrons to quinone (Table 2).

**Table 2:** Distribution of major metabolic pathways and genes among *K. antarctica* strain IMCC3317^T^ and four other *Kordia* strains.

Metabolic pathway and enzyme	*K. antarctica*	*K. algicida*	*K. zhangzhouensis*	*K. jejudonensis*	*K. periserrulae*
**Carbon**					
Glycolysis/gluconeogenesis	O	O	O	O	O
Citrate cycle (TCA cycle)	O	O	O	O	O
Malate dehydrogenase (quinone) [EC:1.1.5.4]	o	x	x	x	x
Pentose phosphate pathway (non-oxidative)	O	O	O	O	O
Glyoxylate cycle	X	O	O	O	O
**Nitrogen**					
Ammonia assimilation	O	O	O	O	O
Glutamine synthetase/glutamate synthase	o	o	o	o	o
Glutamate dehydrogenase (NADP) [EC:1.4.1.4]	o	o	o	o	o
Glutamate dehydrogenase (NAD(P)) [EC:1.4.1.3]	o	x	x	x	x
Nitrous oxide reductase [EC:1.7.2.4]	o	x	x	x	x
**Sulphur**					
Assimilatory sulfate reduction	O	X	X	X	X
Sulfate adenylyltransferase [EC:2.7.7.4]	o	o	o	x	x
Adenylylsulfate kinase [EC:2.7.1.25]	o	x	x	x	x
Phosphoadenylylsulfate reductase (thioredoxin) [EC:1.8.4.8]	o	o	o	x	x
Sulfite reductase (ferredoxin) [EC:1.8.7.1]	o	o	o	x	x
Sulfide:quinone oxidoreductase [EC:1.8.5.4]	x	x	o	x	o
**Phosphorus**					
High-affinity phosphate ABC transporter [EC:7.3.2.1]	x	x	x	x	x
Alkaline phosphatase [EC:3.1.3.1]	o	o	o	o	o

Large and small symbols indicate pathways and genes, respectively (presence, O or o; absence, X or x).

Several differences were found amongst the *Kordia* genomes regarding accessory carbon metabolism. Strain IMCC3317^T^ was predicted to lack a glyoxylate cycle, while the other four strains were equipped with isocitrate lyase (EC 4.1.3.1) and malate synthase (EC 2.3.3.9), two key enzymes for this cycle (Table 2). Strains IMCC3317^T^ and *K. periserrulae* IMCC1412^T^ encoded genes for the Leloir galactose utilization pathway, including galactokinase (EC 2.7.1.6) and galactose-1-phosphate uridylyltransferase (EC 2.7.7.12). Only strain IMCC3317^T^ possessed a lactate dehydrogenase (EC 1.1.1.28; 2713616031), which may facilitate fermentation under anaerobic conditions. All five *Kordia* genomes encoded phosphoenolpyruvate carboxylase (EC 4.1.1.31), an anaplerotic enzyme that converts pyruvate to oxaloacetate via the incorporation of CO_2_. However, pyruvate carboxylase (EC 6.4.1.1), another representative anaplerotic enzyme, was not found in any *Kordia* genomes.

### Nitrogen related metabolism

All five *Kordia* genomes encoded genes for glutamine synthetase and glutamate synthase for ammonia assimilation (Table 2). All genomes possessed two glutamine synthetase genes of different lengths located next to each other in opposite directions. One group of glutamine synthetase genes encoded a type III enzyme of ~ 730 amino acids and the other encoded enzymes of ~ 340 amino acids. In the IMCC3317^T^ genome, genes for an ammonia transporter and nitrogen regulatory protein P-II were located just upstream of glutamate synthase. The five *Kordia* genomes also contained genes for glutamate dehydrogenase (GdhA) responsible for another ammonia assimilation pathway that synthesizes glutamate directly from ammonia. There are three types of GdhA, each showing different cofactor specificities: NAD-dependent (EC 1.4.1.2), NADP-dependent (EC 1.4.1.4), and NAD(P)-dependent (EC 1.4.1.3). All *Kordia* genomes possessed the NADP-dependent type (EC 1.4.1.4), but only the IMCC3317^T^ genome encoded an additional GdhA annotated as a dual cofactor-specific type^27^ (EC 1.4.1.3; 2713618789). Interestingly, a *nosZ* gene was found in the IMCC3317^T^ genome, but not in other *Kordia* genomes. *nosZ* encodes a nitrous oxide reductase that reduces nitrous oxide to nitrogen, the final step in the denitrification pathway. No other genes involved in denitrification were found in the IMCC3317^T^ genome. A more detailed analysis of the *nosZ* gene found in strain IMCC3317^T^ is presented below.

### Sulfur metabolism

Only strain IMCC3317^T^ had a complete assimilatory sulfate reduction pathway wherein sulfate is reduced to sulfide and sequentially incorporated into the biomass, usually via sulfur-containing amino acids such as methionine and cysteine (Table 2). In many bacteria, assimilatory sulfate reduction is accomplished through four steps sequentially mediated by sulfate adenylyltransferase (CysND; EC 2.7.7.4), adenylylsulfate kinase (CysC; EC 2.7.1.25), phosphoadenylylsulfate reductase (thioredoxin) (CysH; EC 1.8.4.8), and sulfite reductase (ferredoxin) (Sir; EC 1.8.7.1). In the genomes of the strains *K*. *algicida*, *K*. *zhangzhouensis*, and *K*. *antarctica*, a homologous gene cluster containing *cysND*, *cysH*, and *sir* genes, but not the *cysC* gene, was found in the vicinity of methionine biosynthesis-related genes. The genomes of *K*. *algicida* and *K*. *zhangzhouensis* did not encode a *cysC* gene, suggesting an incomplete assimilatory sulfate reduction pathway in these strains. In contrast, the IMCC3317^T^ genome encoded a *cysC* gene (2713614888) in another gene cluster that contained an additional copy of the *cysND* gene (2713617813, 2713617814), located at the 5´ region of a non-ribosomal peptide synthetase (NRPS) biosynthetic gene cluster. This finding indicates that only IMCC3317^T^ possesses a complete pathway for assimilatory sulfate reduction, which may confer a competitive advantage when reduced sulfur compounds are scarce. Genes for the Sox pathway of sulfur oxidation that enables sulfur compound utilization as an energy source were not predicted for any of the *Kordia* genomes. However, a sulfide:quinone oxidoreductase that mediates the electron transfer from sulfide to the quinone pool was found in the *K*. *zhangzhouensis* (2628586580) and *K*. *periserrulae* genomes (2735934522) (Table 2).

### Phosphorus metabolism

Regarding phosphorus metabolism, strain IMCC3317^T^ and other *Kordia* strains appear to specialize in the utilization of organophosphate but not inorganic phosphate. Many representative marine bacterial groups abundant in pelagic ocean, such as the SAR11 clade and *Prochlorococcus*, possess a high-affinity phosphate ABC transporter (EC 7.3.2.1; PstSCAB), which may be an adaption to phosphate-depleted oligotrophic pelagic waters. However, all *Kordia* genomes lacked this ABC transporter, implying that the genus *Kordia* has not adapted to phosphate-depleted oligotrophic condition. Instead, all *Kordia* genomes encoded genes for alkaline phosphatase D (PhoD) and PhoPR, a two-component signal transduction system. Alkaline phosphatases (EC 3.1.3.1) can liberate phosphate from organic phosphorus compounds via hydrolysis of phosphoester bonds. Among the three prokaryotic alkaline phosphatase groups known to date, PhoA, PhoX, and PhoD, PhoD was more abundant than the other two groups among global ocean sampling (GOS) metagenomes^28^. Given that all *Kordia* PhoD proteins contain signal peptides, it is likely that *Kordia* strains secrete PhoD into the periplasm or extracellular milieu and subsequently uptake the phosphate released from dissolved organic phosphorus compounds. The possession of secretable alkaline phosphatase suggests that the genus *Kordia* may be adapted to coastal waters replete with organophosphate compounds. Whether and how the expression of PhoD is regulated by the PhoPR system in the *Kordia* strains, however, remains unclear.

### Secondary metabolites

Analyses by antiSMASH showed that the IMCC3317^T^ genome contained nine putative biosynthetic gene clusters (BGCs) with lengths ranging from 21 to 225 kb. These BGCs included a range of clusters, such as NRPS, T1PKS [Type I polyketide synthase (PKS)], T3PKS (Type III PKS), transAT-PKS (Trans-AT PKS), CDPS (tRNA-dependent cyclodipeptide synthase), arylpolyene, lanthipeptide, and terpene. Considering that BGCs are found rarely in marine bacterial groups that have streamlined genomes and are abundant in pelagic waters (e.g. the SAR11 clade), the possession of multiple BGCs that can produce secondary metabolites may be an adaptation to coastal waters where the interactions with other organisms are expected to be more active than pelagic ocean.

The longest BGC, spanning ~ 225 kb (802,427–1,027,059 bp), was identified as a polyketide-NRPS hybrid gene cluster and predicted to synthesize a polymer consisting of at least 30 amino acids (Suppl. Fig. S1). The antiSMASH results showed that gene clusters similar to this BGC were found in certain flavobacterial strains, such as *Flavobacterium spartansii* MSU, *Flavobacterium chilense* DSM 24724, *Flavobacterium johnsoniae* UW101, *Flavobacterium* sp. WG21, and *Kordia zhangzhouensis* MCCC 1A00726, suggesting wide distribution among the family *Flavobacteriaceae*. Of note, however, none of these gene clusters have been fully characterized, leading to a low similarity between this *K*. *antarctica* BGC and those curated in the MIBiG, a database of BGCs and their products. This *K*. *antarctica* BGC shared only 3% to 4% of its genes with the most similar BGCs found in the MIBiG database, and there was no homology with the core biosynthetic genes of this BGC, indicating the *K*. *antarctica* BGC and similar BGCs found in other flavobacterial genomes might synthesize novel secondary metabolites.

### Carbohydrate-active enzymes (CAZymes)

Many CAZymes and SusCD proteins were found in the IMCC3317^T^ genome, suggesting a metabolic potential for polysaccharide utilization. Members of the marine *Flavobacteriaceae* specialize in degradation and utilization of polysaccharides produced primarily by algae and phytoplankton^3,29^, making this heterotrophic bacterial group a major contributor to the turnover of dissolved or particulate high-molecular weight organic matter^30^. Recent studies on genomics, transcriptomics, and proteomics of representative marine flavobacterial strains showed that a diverse set of proteins, including transporters, CAZymes, and sulfatases, are critical to polysaccharide metabolism; moreover, the genes encoding these proteins are usually gathered into clusters termed polysaccharide utilization loci (PULs)^8,31^.

**Figure 4:**
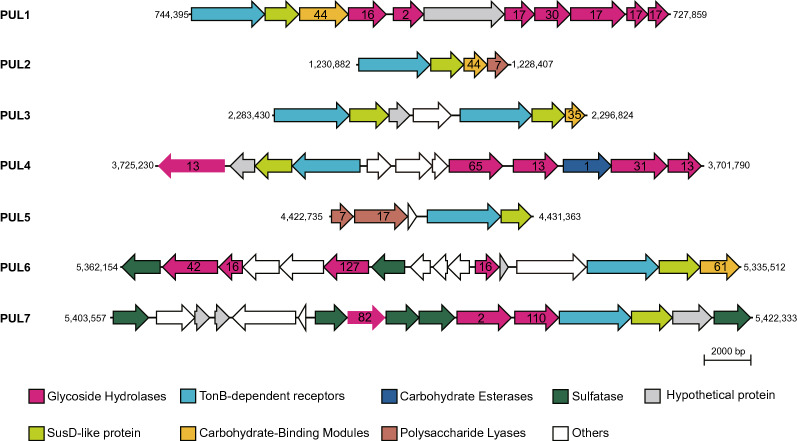
Seven polysaccharide utilization loci (PUL) of *K. antarctica* IMCC3317^T^ predicted based on eight SusCD pairs and adjacent CAZymes. Genes are colored according to function (color key at bottom). Families of CAZyme genes indicated by numbers within arrows. Numbers on either side of each PUL map indicate start and end positions in the genome. 'Others' refers to annotated genes with functions other than CAZyme, SusCD, and sulfatase.

As such, we screened and analyzed PULs from the IMCC3317^T^ genome, focusing on CAZymes and SusCD, the two major constituents of PULs, to reveal the genomic potential of marine flavobacterial strain IMCC3317^T^ for polysaccharide metabolism. The *susC* and *susD* genes, located adjacently in many PULs, encode a TonB-dependent transporter and carbohydrate-binding lipoprotein, respectively. SusC and SusD form a complex in the outer membrane to bind and uptake carbohydrates^32^. CAZymes found in PULs include glycoside hydrolases (GH), glycosyltransferases (GT), carbohydrate esterases (CE), and polysaccharide lyase (PL), and are involved in the further breakdown and metabolism of various carbohydrates. Analysis of the IMCC3317^T^ genome using dbCAN showed a total of 194 putative CAZyme proteins, including 53 GH (20 families), 57 GT (12 families), 36 CE (8 families), nine PL (5 families), and 45 carbohydrate-binding modules (14 families). PFAM and TIGRFAM-based searches for SusC and SusD revealed that the IMCC3317^T^ genome possessed 10 SusCD pairs. An inspection of genomic regions around these pairs showed that eight SusCD pairs constituted seven PULs together with neighboring CAZymes (Fig. 4). GntR family transcriptional regulators or sulfatases were also found within many PULs. One of the PULs (PUL1, Fig. 4) contained GH families 16, 17, and 30, suggesting the utilization of laminarin^8^. Collectively, the IMCC3317^T^ genome has many genes necessary for polysaccharide utilization, which may be regarded as an adaptation to coastal seas where phytoplankton and algae may produce a large amount of polysaccharides.

### Nitrous oxide reductase gene (*nosZ*)

A *nosZ* gene (2713616369) encoding nitrous oxide reductase was found in the IMCC3317^T^ genome, but not in the other *Kordia* genomes. The NosZ enzyme localizes to the periplasmic space and is involved in the final step of denitrification; that is, the reduction of nitrous oxide (N_2_O) to nitrogen gas (N_2_)^33^. According to a recent phylogenetic analysis^37^, there are two distinct clades of NosZ, each with different signal peptides related to different translocation pathways. While the clade I NosZ localizes to the periplasm via the twin-arginine translocation pathway, the clade II NosZ is transported via the Sec-dependent translocation pathway. An analysis of signal peptides showed that the NosZ of strain IMCC3317^T^ belongs to clade II. Since no other enzymes involved in the denitrification pathway (such as nitrate reductase and nitrite reductase) were found in the IMCC3317^T^ genome, like many other prokaryotes with clade II NosZ, IMCC3317^T^ was designated as a non-denitrifying N_2_O reducer. It has been suggested that phylogenetically diverse non-denitrifying N_2_O reducers with a clade II NosZ may be important N_2_O sinks since these organisms can consume, but not produce, N_2_O in the environment^34^. From a climate change perspective, given that N_2_O is a major greenhouse gas, at around 300 times more warming potential than CO_2_ and implicated in ozone depletion, the existence of non-denitrifying N_2_O reducers, such as IMCC3317^T^, is critical for the conversion of N_2_O to harmless N_2_^35^.

**Figure 5:**
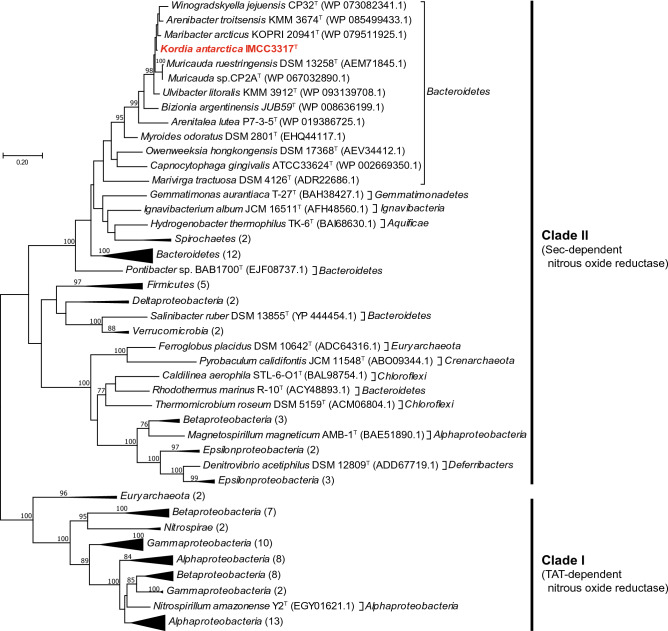
Maximum-likelihood phylogenetic tree of NosZ proteins from different bacterial groups. NosZ of IMCC3317^T^ is bolded in red. Designation of NosZ clades I and II based on Hallin et al.^37^. Phylogenetic affiliation at phylum/class level indicated after brackets to the right of taxa names. For visualization purposes, certain NosZ genes from the same phylum/class were grouped together and described as wedges, with the number of grouped proteins indicated in parentheses following phylum/class name. Bootstrap values (≥ 70; 100 replicates) indicated above the branches.

The *nosZ* gene was found only in IMCC3317^T^_,_ and analyses were thereby performed to infer the phylogenetic position of the IMCC3317^T^ NosZ. In a BLASTp search against the NCBI nr database, top hits for IMCC3317^T^ NosZ were predominantly from marine flavobacterial genera phylogenetically related to *Kordia*. The IMCC3317^T^ NosZ sequence and several best BLASTp hits were used for the construction of maximum-likelihood trees together with representative NosZ sequences^36,37^ and IMCC3317^T^ NosZ, and its best BLASTp hits formed a robust branch within the *Bacteroidetes* group of clade II NosZ (Fig. 5). These results suggest that IMCC3317^T^ NosZ is similar to that of other phylogenetically-related flavobacterial genera, notwithstanding the absence of NosZ in other species of the same genus. This finding is consistent with previous research indicating that NosZ phylogeny was predominantly shaped by vertical transfer, and gene gain or loss has led to a variable distribution of NosZ even among closely related organisms^37,38^. Further analyses of the family *Flavobacteriaceae* genomes (available in the IMG database) revealed that the variable distribution of NosZ among the same genera was not limited to *Kordia*, but widespread throughout the family *Flavobacteriaceae*. Many genera, including *Tenacibaculum*, *Maribacter*, *Muricauda*, *Aquimarina*, and *Winogradskyella*, had both NosZ-possessing genomes and NosZ-lacking genomes (Suppl. Table S1). More elaborate analyses of the phylogenomics of flavobacterial strains and phylogeny of their NosZ proteins will be necessary to reconstruct the evolutionary history of NosZ in the family *Flavobacteriaceae* and provide a plausible explanation for the distribution of NosZ in *Kordia* among the following two scenarios: all *Kordia* strains, except for *K. antarctica* IMCC3317^T^, have lost the *nosZ* gene inherited from the common ancestor of the genus *Kordia*; or, the common *Kordia* ancestor lacked the *nosZ* gene and strain IMCC3317^T^ acquired it via horizontal gene transfer from closely related organisms.

In many *nosZ*-harboring genomes, *nosZ* is clustered with other genes involved in protein assembly and transport of copper, a cofactor of NosZ. The *nosZ* gene of IMCC3317^T^ is located within a gene cluster arranged as *nosZ*, *nosL* (copper chaperone; 2713616367), *nosD* (accessory protein; 2713616366), *nosF* (Cu-processing system ATP-binding protein; 2713616365), and *nosY* (Cu-processing system permease protein; 2713616364). This gene order is highly conserved among *nosZ*-possessing flavobacterial strains closely related to the genus *Kordia*.

## Conclusion

The genome sequence of *K. antarctica* IMCC3317^T^ reported in this study represents the first complete, published genome of the genus *Kordia*. Analyses of this complete genome showed that strain IMCC3317^T^ shared certain chemoheterotroph metabolic features with other *Kordia* strains. Also, like the two *Kordia* genomes (*K*. *algicida* and *K*. sp. SMS9) listed in PULDB^39^, the IMCC3317^T^ genome possessed many PULs, suggesting the utilization of polysaccharides originated by marine phytoplankton or algae. Based on the prediction of a *nosZ* gene in the IMCC3317^T^ genome in the absence of other denitrification genes, strain IMCC3317^T^ is considered a non-denitrifying N_2_O-reducing bacteria. Further studies will be required to explore whether strain IMCC3317^T^ can actually reduce N_2_O, a significant greenhouse gas in climate change.

## Methods

### Genome sequencing

Strain IMCC3317^T^ was resuscitated by incubating a loopful of 10% glycerol stock on marine agar 2216 plates at 20℃ for 7 days. Genomic DNA was extracted from colonies grown on these plates with a DNeasy Blood & Tissue Kit (Qiagen, Hilden, Germany) according to the manufacturer’s protocol. Genome sequencing of IMCC3317^T^ was conducted on the PacBio RS II platform (Pacific Bioscience, Menlo Park, CA, USA) with a 20 kb SMRTbell library generation. De novo assembly was conducted with the SMRT Analysis RS_HGAP_Assembly.2 protocol (v2.3.0) using ~ 38,000 filtered reads (~ 583 Mbp total; N50 read length, 20,089 bp), resulting in a single contig with approximately ~ 93 × coverage. This single contig was circularized using Circlator (v1.5.5; ‘all’ command with default options)^40^ and polished five times with the SMRT Analysis RS_Resequencing.1 protocol until no variants were called, generating a final, error-corrected genome sequence. The GenBank accession number of the complete IMCC3317^T^ genome is CP019288.

### Genome annotation and comparison

The complete genome sequence of IMCC3317^T^ was submitted to the IMG-ER system^41^ for detailed annotation and comparative analyses. Prokka (v1.12)^42^ was also used for local annotation (with the options ‘--rfam’ and ‘--rnammer’). Protein coding sequences, ribosomal RNA genes, transfer RNA genes, and non-coding RNAs were predicted using the tools (implemented in Prokka) Prodigal (v2.6.3), RNAmmer (v1.2), ARAGORN (v1.2), and Infernal (v1.1), respectively. The GenBank file produced by Prokka was submitted to the RAST server for improved functional annotation^43^ with classic RAST annotation scheme and ‘preserve original genecalls’ option. Where necessary, annotation information from Prokka and the RAST server were used for comparison with IMG-ER annotation. HMMs from dbCAN^44^ were used to predict and classify CAZymes with default parameters. SusCD proteins were predicted based on the presence of PFAM (for SusD; PF07980, PF12741, PF12771, and PF14322) and TIGRFAM (for SusC; TIGR04056 and TIGR04057) domains. Biosynthetic gene clusters for secondary metabolites were predicted using antiSMASH 5.0^45^ with default settings.

Comparative analyses with four other *Kordia* genomes were generally performed using the functionalities provided by the IMG-ER system. All four genomes included in comparative analyses were from the type strains of the genus *Kordia* available in the IMG database: *K*. *algicida* OT-1 (IMG genome ID; 641380434), *K*. *zhangzhouensis* MCCC 1A00726 (2627853697), *K*. *jejudonensis* SSK3-3 (26364156330), and *K*. *periserrulae* DSM 25731 (2734482288). The completeness of the genome sequences was calculated using CheckM (v1.1.2) with default setting^46^. Average nucleotide identity (ANI) values between genomes were calculated using JSpeciesWS^47^. Strain genome sequences were downloaded and used to draw a plot for genomic comparison between the *K*. *antarctica* IMCC3317^T^ genome and the other four *Kordia* genomes using BRIG^48^. Protein sequences were also downloaded and used for the clustering of homologous proteins among the five *Kordia* genomes by OrthoFinder (v2.2.7)^49^ at default settings, and a Venn diagram showing the number of shared or unique protein clusters was generated using the R packages ‘limma’ and ‘gplot’. Protein sequences were also analyzed by BlastKOALA (ran on August 13, 2018)^50^ to assign them to KEGG orthology (KO) groups and reconstruct metabolic pathways. A comparative analysis of metabolic pathways was conducted using the KO ID, EC number, COG ID, and Pfam ID of major metabolic enzymes of five *Kordia* genomes in searches against multiple databases, including MetaCyc. When necessary, protein sequences were searched against the NCBI nr database.

### Phylogenetic analysis of NosZ protein

Protein sequences highly similar to that of the NosZ protein found in the IMCC3317^T^ genome were searched for and retrieved from the NCBI RefSeq database using BLASTP 2.8.1+ (ran on September 11, 2018). These sequences were used for phylogenetic analyses together with 109 representative NosZ proteins selected based on previous studies on the distribution and phylogeny of NosZ^37,51^. The collected protein sequences were aligned using Muscle and the aligned sequences were then used for a tree building by the Maximum Likelihood method based on the JTT model using MEGA7^52^.

We analyzed the distribution of the *nosZ* gene among members of *Flavobacteriaceae* using genomes publicly available in the IMG database (accessed at Oct, 2018). Genomes classified as *Flavobacteriaceae* in IMG were analyzed using the Function Profile utility. Any genome harboring gene(s) assigned to K00376, a KEGG ortholog corresponding to nitrous oxide reductase (NosZ), was considered to contain a *nosZ* gene. Single-cell genomes, metagenome-assembled genomes, and genomes from bacterial isolates not classified at the genus level were excluded from the analysis.

## Supplementary information

Supplementary information.
