# Supplementary material for: Genome characteristics of Kordia antarctica IMCC3317T and comparative genome analysis of the genus Kordia
Source: Sci Rep. 2020 Sep 7;10:14715. doi: 10.1038/s41598-020-71328-9 (PMC7477175; doi:10.1038/s41598-020-71328-9)
Supplement: Supplementary file 1 — Supplementary information. [file 41598_2020_71328_MOESM1_ESM.pdf]

Supplementary Material

Genome characteristics of *Kordia antarctica* IMCC3317^T and comparative genome analysis of the genus *Kordia*

Yeonjung Lim[1], Ilnam Kang[2 *], Jang-Cheon Cho[1 *]

[1] Department of Biological Sciences, Inha University, Incheon 22212, Republic of Korea

[2] Center for Molecular and Cell Biology, Inha University, Incheon 22212, Republic of Korea

***Corresponding authors**:

Jang-Cheon Cho, Department of Biological Sciences, Inha University, Incheon 22212, Republic of Korea, chojc@inha.ac.kr

Ilnam Kang, Center for Molecular and Cell Biology, Inha University, Incheon 22212, Republic of Korea, ikang@inha.ac.kr

Supplementary Table 1. Distribution of *nosZ* gene in *Flavobacteriaceae*.

Genus	Number of genomes	Number of genomes having *nosZ*	Percentage (%)
Flavobacterium	193	41	21.2
Chryseobacterium	136	9	6.6
Capnocytophaga	47	12	25.5
Elizabethkingia	37	0	0.0
Tenacibaculum	28	6	21.4
Myroides	24	13	54.2
Maribacter	24	9	37.5
Polaribacter	19	1	5.3
Nonlabens	19	0	0.0
Muricauda	16	13	81.3
Cellulophaga	15	3	20.0
Riemerella	14	14	100.0
Aquimarina	14	6	42.9
Winogradskyella	14	5	35.7
Aequorivita	12	12	100.0
Salegentibacter	12	11	91.7
Arenibacter	11	11	100.0
Gramella	11	3	27.3
Formosa	10	3	30.0
Lacinutrix	9	4	44.4
Olleya	9	4	44.4
Lutibacter	8	4	50.0
Gillisia	8	3	37.5
Algibacter	8	1	12.5
Dokdonia	8	0	0.0
Leeuwenhoekiella	7	0	0.0
Psychroflexus	6	5	83.3
Salinimicrobium	5	5	100.0
Psychroserpens	5	3	60.0
Kordia	5	1	20.0
Croceibacter	5	0	0.0
Mesonia	5	0	0.0
Zunongwangia	5	0	0.0
Bizionia	4	2	50.0
Ulvibacter	4	2	50.0
Jejuia	4	1	25.0
Mangrovimonas	4	1	25.0
Bergeyella	4	0	0.0
Ornithobacterium	4	0	0.0
Tamlana	4	0	0.0
Gaetbulibacter	3	3	100.0
Gelidibacter	3	3	100.0
Xanthomarina	3	3	100.0
Flagellimonas	3	2	66.7
Zobellia	3	2	66.7
Confluentibacter	3	0	0.0
Empedobacter	3	0	0.0
Pseudozobellia	3	0	0.0

Genus	Number of genomes	Number of genomes having *nosZ*	Percentage (%)
Weeksella	3	0	0.0
Altibacter	2	2	100.0
Robiginitalea	2	2	100.0
Zhouia	2	2	100.0
Mesoflavibacter	2	1	50.0
Seonamhaeicola	2	1	50.0
Wenyingzhuangia	2	0	0.0
Arenitalea	1	1	100.0
Aureitalea	1	1	100.0
Cloacibacterium	1	1	100.0
Eudoraea	1	1	100.0
Flavivirga	1	1	100.0
Hanstruepera	1	1	100.0
Hyunsoonleella	1	1	100.0
Ichthyenterobacterium	1	1	100.0
Imtechella	1	1	100.0
Kriegella	1	1	100.0
Mariniflexile	1	1	100.0
Muriicola	1	1	100.0
Pricia	1	1	100.0
Vitellibacter	1	1	100.0
Zeaxanthinibacter	1	1	100.0
Algoriella	1	0	0.0
Apibacter	1	0	0.0
Chishuiella	1	0	0.0
Croceitalea	1	0	0.0
Croceivirga	1	0	0.0
Cruoricaptor	1	0	0.0
Flaviramulus	1	0	0.0
Galbibacter	1	0	0.0
Joostella	1	0	0.0
Marinirhabdus	1	0	0.0
Meridianimaribacter	1	0	0.0
Moheibacter	1	0	0.0
Oceanihabitans	1	0	0.0
Pustulibacterium	1	0	0.0
Sediminibacter	1	0	0.0
Sediminicola	1	0	0.0
Siansivirga	1	0	0.0
Sinomicrobium	1	0	0.0
Soonwooa	1	0	0.0
Vaginella	1	0	0.0

Genomes classified as the *Flavobacteriaceae* in the IMG database (accessed at December, 2018) were analyzed. If a genome has gene(s) assigned to K00376, a KEGG ortholog corresponding to nitrous oxide reductase (NosZ), the genome was regarded to have nosZ gene.

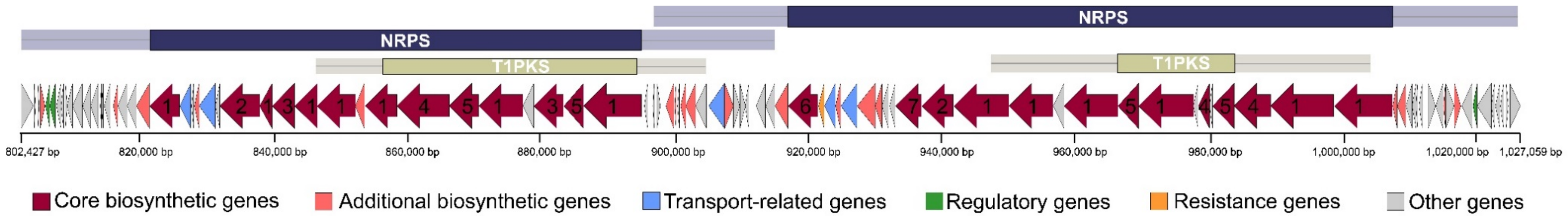

Supplementary Figure 1. A gene map showing the longest BGC (biosynthetic gene cluster) found in the IMCC3317^T genome that was predicted to be a polyketide-NRPS hybrid gene cluster. Genes are indicated in color according to the function categories at the bottom. Core biosynthetic genes are numbered following their specific annotations: 1, tyrocidine synthase 3; 2, surfactin synthase subunit; 3, plipastatin synthase subunit; 4, linear gramicidin synthase subunit; 5, phthiocerol/phenolphthiocerol synthesis polyketide synthase type I PpsE; 6, dimodular nonribosomal peptide synthase; 7, gramicidin S synthase.
